# Understanding norovirus reporting patterns in England: a mixed model approach

**DOI:** 10.1186/s12889-021-11317-3

**Published:** 2021-06-28

**Authors:** N. Ondrikova, H. E. Clough, N. A. Cunliffe, M. Iturriza-Gomara, R. Vivancos, J. P. Harris

**Affiliations:** 1 Institute of Infection, Veterinary and Ecological Sciences, University of Liverpool, Liverpool, UK; 2 Institute for Risk & Uncertainty, University of Liverpool, Liverpool, UK; 3 NIHR Health Protection Unit in Gastrointestinal Infections, University of Liverpool, Liverpool, UK; 4 Public Health England, Liverpool, UK; 5 Centre for Vaccine Innovation and Access, PATH, Geneva, Switzerland; 6 NIHR Health Protection Unit in Emerging and Zoonotic Infections, University of Liverpool, Liverpool, UK

**Keywords:** Norovirus, HHH4, Underestimation, Public health surveillance, Mixed-effects, Negative binomial

## Abstract

**Background:**

Norovirus has a higher level of under-reporting in England compared to other intestinal infectious agents such as *Campylobacter* or *Salmonella*, despite being recognised as the most common cause of gastroenteritis globally. In England, this under-reporting is a consequence of the frequently mild/self-limiting nature of the disease, combined with the passive surveillance system for infectious diseases reporting. We investigated heterogeneity in passive surveillance system in order to improve understanding of differences in reporting and laboratory testing practices of norovirus in England.

**Methods:**

The reporting patterns of norovirus relating to age and geographical region of England were investigated using a multivariate negative binomial model. Multiple model formulations were compared, and the best performing model was determined by proper scoring rules based on one-week-ahead predictions. The reporting patterns are represented by epidemic and endemic random intercepts; values close to one and less than one imply a lower number of reports than expected in the given region and age-group.

**Results:**

The best performing model highlighted atypically large and small amounts of reporting by comparison with the average in England. Endemic random intercept varied from the lowest in East Midlands in those in the under 5 year age-group (0.36, CI 0.18–0.72) to the highest in the same age group in South West (3.00, CI 1.68–5.35) and Yorkshire & the Humber (2.93, CI 1.74–4.94). Reporting by age groups showed the highest variability in young children.

**Conclusion:**

We identified substantial variability in reporting patterns of norovirus by age and by region of England. Our findings highlight the importance of considering uncertainty in the design of forecasting tools for norovirus, and to inform the development of more targeted risk management approaches for norovirus disease.

**Supplementary Information:**

The online version contains supplementary material available at 10.1186/s12889-021-11317-3.

## Background

Norovirus is recognised as the most common cause of diarrhoeal disease globally [1] but has the highest levels of under-reporting compared to other intestinal infectious agents such as *Campylobacter* or *Salmonella* [2]. In England, the level of this under-reporting is a consequence of the nature of the disease and the surveillance system for infectious diseases. The illness is characterised by a sudden onset of symptoms and is generally self-limiting, lasting around two to 3 days in otherwise healthy individuals, and most people recover without contacting medical services. However, some individuals suffer more severe disease outcomes in [3, 4]. Norovirus is not a notifiable disease in England. However, there is a statutory duty on the providers of diagnostic laboratory services to report to Public Health England (PHE) isolates of an infectious agent within 7 days [5] and norovirus is often reported this way. Reporting of outbreaks in care homes and in health care settings is encouraged by the regulator (Care Quality Commission) in England, but it remains voluntary. All routine laboratory reports of norovirus are reported to the national laboratory surveillance system – Second-Generation Surveillance System (SGSS) [6].

Norovirus places a considerable burden on health care services in England both financial, with the cost estimated at between £63 and £106 million annually [7], and in terms of capacity adding significantly to the annual “winter pressures”. To reduce this burden, PHE recommends that affected individuals stay at home until symptoms have resolved. While not seeking medical attention prevents norovirus from spreading, it limits the chances of isolated cases to feature in a national surveillance system [8]. Consequently, the surveillance system might be prone to represent cases associated with outbreaks particularly from semi-closed settings such as care homes and hospitals rather than those in the community [9].

A better understanding of the heterogeneity in passive surveillance system improves understanding of differences in reporting and laboratory testing practices across geographic regions and age groups. Statistical modelling approaches can help quantify this heterogeneity. Count data are often modelled using a Poisson distribution: however biological data commonly exhibit greater variation than the Poisson model can accommodate (called “over-dispersion”). The negative binomial distribution is a viable alternative. To account for heterogeneity due to individual or regional differences, random effects are commonly used. However, the use of random effects brings challenges when it comes to identifying the best model formulation. Typically, Akaike (AIC) or Bayesian information criterion (BIC) is used. When the model contains random effects the definition of the AIC and BIC is not straightforward [10], and the use of proper scoring rules is one approach to overcome these difficulties [11]. This approach has been described elsewhere [12]. Briefly, the approach assesses predictive distribution based on predictions from the proposed model.

We investigated the reporting patterns of norovirus relating to geographical region and age in England. A simpler analysis of earlier data which motivated the current study is reported in [13]. First, an age-stratified multivariate discrete spatio-temporal model was fitted. Then, we incorporated random effects into four model formulations which were evaluated against the simpler model and each other. This led to the selection of the best performing model based on one-week-ahead predictions. Finally, we used the best performing model to highlight regions with atypically large or small amounts of reporting by comparison with the average in England. This information is highly relevant for public health policy and planning but also for any research using routine data.

## Methods

### Data

All diagnostic laboratories in England report data to the SGSS. The process of data validation and management is described elsewhere, e.g. [6]. We obtained the weekly numbers of laboratory confirmed norovirus cases between week 27, 2014 (June) and week 26, 2019 (July) for nine regions of England from SGSS stratified by age. The period did not coincide with an emergence of a new strain; the norovirus Sydney2012 strain was dominant throughout the study [14].

The Office for National Statistics (ONS) provides age-stratified population estimates for England. We obtained regional population data stratified by six age groups: 0–4, 5–14, 15–24, 25–44, 45–64, 65+. Social contacts matrix for these age groups is based on physical as well as non-physical contacts of UK subset of the POLYMOD study [15].

We also obtained the numbers of primary schools and nurseries [16] and hospitals [17] in each region, since norovirus is known to cause outbreaks in closed and semi-closed environments. These regional counts were normalised to range from 0 to 1 and thus providing regional proportions.

The R code to import and prepare data for modelling is provided in public GitHub repository (see Availability of data and materials).

### Statistical modelling

The multivariate time-series modelling framework allows for additive decomposition of aggregated time series into endemic and epidemic components representing origins of an infection spread [11, 18, 19]. The initial formulation described in Held et al. [18] was later extended to consider social contacts within a population [19], and heterogeneity was often found in count data [11]. We adopted these methods to investigate reporting patterns of norovirus in England, but the terminology is inherited.

The endemic component represents sporadic cases: in other words, the number of reported norovirus cases that would be expected in specific regions and age-groups in the absence of outbreaks. The epidemic component conceptually represents reported cases emerging from outbreaks. The spatial and temporal spread is specified in terms of power-law distance decay. One of the power-law decay benefits is the relaxation of a simple assumption that the epidemic can only spread to a directly neighbouring region, i.e. a region with a shared border. Moreover, when distance decay depends on the population, it can describe temporal (i.e. within-region) spread, often referred to as a gravity model, e.g. [20].

Weekly count of norovirus cases *(t = 1, …, 52)* per age group *(g = 1, …, 6)* per region of England *(r = 1, …, 9)* is denoted by *Y*_*grt*_. Then, conditional upon previous observations, *Y*_*grt*_ is assumed to follow a negative binomial distribution with mean

1 $$ {\mu}_{gr t}={pop}_{gr}{\nu}_{gr t}+{\phi}_{gr t}{\sum}_{g`r`}\ \left\lfloor {C}_{g`g}{W}_{r`r}\right\rfloor\ {Y}_{g`,r`,t-1} $$

The population fraction *pop*_*gr*_ based on mid-2016 OSN estimates is used as an offset for the endemic component *ν*_*grt*_. Since we were interested in reflecting the differences in population size across age groups and geographic units rather than temporal variation in the population per se, temporal variation in population data was not explicitly modelled. The row-normalised product of POLYMOD contact matrix *C*_*g* ‘ *g*_ and spatial weights *W*_*r* ‘ *r*_ summed over the age group *g’* and region *r’* forms the epidemic component *ϕ*_*grt*._ In other words, the product determines how the counts from the previous period affect the current mean in the age group and region [19]. Mathematical expression of *ν*_*grt*_ and *ϕ*_*grt*_ in fixed-effect formulations from the previous equation are

2 $$ \log \left({\nu}_{grt}\right)={\alpha}_0^{\left(\nu \right)}+{\alpha}_g^{\left(\nu \right)}+{\alpha}_1{x}_{1r}^{\left(\nu \right)}+{\alpha}_2{x}_{2r}^{\left(\nu \right)}+{\alpha}_3{x}_{3r}^{\left(\nu \right)}+{\beta}_t+{\sum}_{s=2}^s\left\{{\gamma}_s^{\left(\nu \right)}\sin \left({\omega}_st\right)+{\delta}_s^{\left(\nu \right)}\cos \left({\omega}_st\right)\right\} $$

3 $$ \log \left({\phi}_{gr t}\right)={\alpha}_0^{\left(\phi \right)}+{\alpha}_g^{\left(\phi \right)}+\tau log\left({pop}_{gr}\right)+{\sum}_{s=0}^s\left\{{\gamma}_s^{\left(\phi \right)}\sin \left({\omega}_st\right)+{\delta}_s^{\left(\phi \right)}\cos \left({\omega}_st\right)\right\} $$

We included age-specific fixed effects α_g_ in both components to account for age-related susceptibility, linear trend *β*_*t*_ and *S* number of harmonic waves in curly brackets where the *γ*_*s*_ and *δ*_*s*_ signify seasonal parameters and *ω*_*s*_ = 2*πs*/52 represents Fourier frequencies for weekly data. Additionally, logged regional proportions of number of primary schools ${\alpha}_1{x}_{1r}^{\left(\upnu \right)}$, nurseries ${\alpha}_2{x}_{2r}^{\left(\upnu \right)}$ and hospitals ${\alpha}_3{x}_{3r}^{\left(\upnu \right)}$ were included as endemic covariates. For the rest of the model formulations, random effects ${b}_{gr}^{(.)}$ are added to capture any remaining heterogeneity; ${b}_{gr}^{\left(\nu \right)}\sim N\left(0,{\sigma}_{\nu}^2\right),{b}_{gr}^{\left(\phi \right)}\sim N\left(0,{\sigma}_{\phi}^2\right)$ . These can be correlated or uncorrelated.

As previously mentioned, comparing models employing random effects can be challenging. As recommended by Paul and Held in [11], we use strictly proper scoring rules, namely ranked probability score (RPS) and logarithmic score (logS). The earlier is less sensitive to extreme values, whereas the latter will more strictly penalise them. In other words, logS is more sensitive to a misprediction in outbreak period than RPS. Both scores were calculated from one-week-ahead predictions based on unseen data from norovirus season 2018–2019. The train and test partitions are illustrated in Additional file 1. Model selection is then based on the scores and permutation tests, determining whether one score is significantly better than the other.

The analyses were conducted using R software [21]. Social contacts matrix was obtained from the R package ‘socialMixer’ [15], and modelling tasks were performed with R packages ‘surveillance’ [22] and ‘hhh4contacts’ [19] (Eqs. 1). The penalised log-likelihood in the hhh4 function is penalised using the quasi-Newton algorithm by default. In the case of the mixed-effects models, Nelder-Mead penalisation was preferred to maximise the marginal likelihood concerning the variance parameters [11]. The regression parameters were optimised on the log-scale.

## Results

Initially, we compared fixed-effect models with one (S = 1), two (S = 2) and three (S = 3) seasonal waves in endemic component to determine the baseline model formulation (see Additional file 2). The predictive performance was not significantly different between these models and so two seasonal waves were selected as the baseline.

Table 1 shows that all the models were well-calibrated, as suggested by *p* > .05. A model is well-calibrated when its predictive distribution covers the observed value; for example, when the prediction for week 15 in South West England is 200 cases and the upper bound of the predictive distribution is 190 cases, miscalibration is suspected. Models including random effects with harmonic waves in the epidemic component achieved the lowest scores.

**Table 1:** Performance evaluation of selected models based on proper scoring rules

Model	RPS		logS	
*Endemic seasonality (S = 2) + linear trend + covariates*	Mean score	Calibration test (*p*-Value)	Mean score	Calibration test (*p*-Value)
*No random effects:*				
A1 epidemic (S = 0)	0.938	0.385	1.480	0.270
A2 epidemic (S = 1)	0.934	0.251	1.480	0.177
*Uncorrelated random effects:*				
B1 epidemic (S = 0)	0.890	0.888	1.430	0.499
B2 epidemic (S = 1)	0.884	0.514	1.430	0.245
*Correlated random effects:*				
C1 epidemic (S = 0)	0.890	0.810	1.430	0.448
C2 epidemic (S = 1)	0.884	0.452	1.430	0.211

The two best performing models, B2 and C2, were selected for permutation test-based comparison. The comparison of error scores showed no difference between models in both, RPS (*p* = 0.408) and logS (*p* = 0.067). The B2 model showed lower score but as the models were not significantly different in the predictive performance the second-best model could have been selected as well.

**Fig. 1:**
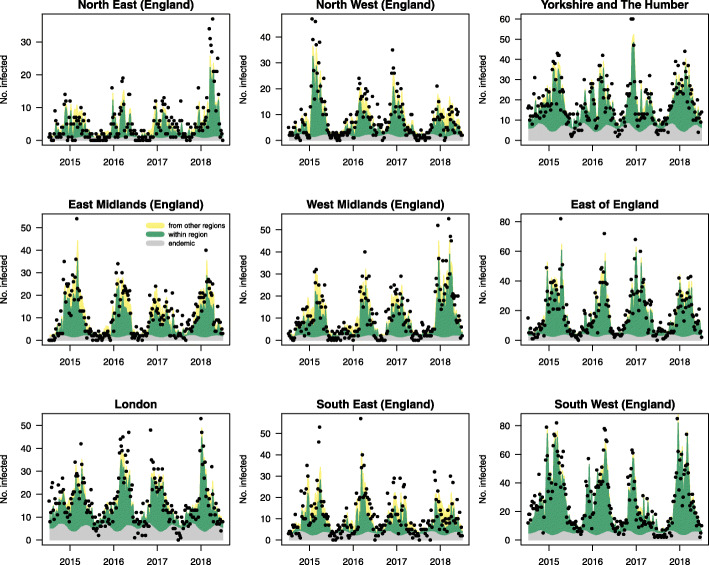
Regional model fit and the relative contribution of model components. Epidemic (within + between) and endemic components per region, aggregated over age groups from the best performing model (B2). The dots represent the actual reported norovirus cases

**Fig. 2:**
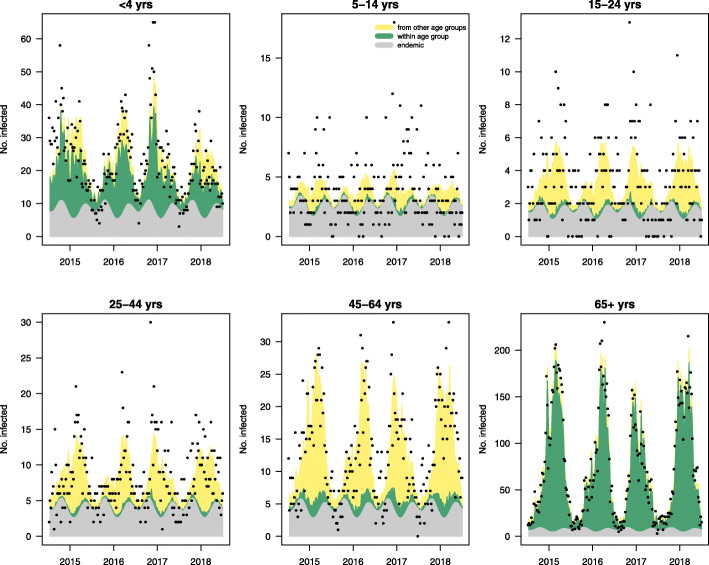
Age-group-based model fit and the relative contribution of model components. Epidemic (within + between) and endemic components per age group, aggregated over regions from the best performing model (B2). The dots represent the actual reported norovirus cases

The point estimates, confidence intervals (CI) and standard errors of the best performing negative binomial regression model with uncorrelated random effects and epidemic seasonal component are reported in Table 2. Considering fixed age-group coefficients, the higher epidemic intercept for the 65+ group (1.796, CI 1.085–2.971) compared to the other groups and the endemic intercept in the same group suggests there is a bias towards reporting of outbreak-generated cases from care homes and hospitals. Generally, the models without epidemic seasonality (B1, C1) suggest that 87% of the reported cases originate from outbreaks, and 13% are endemic in nature. However, models considering seasonal waves (B2, C2) in epidemic component showed that the proportion of outbreak-related reporting is lower in summer (57%). The relative contribution of endemic and epidemic components per region and age group is illustrated in Figs. 1 and 2 respectively. Figure 2 also shows high levels of within-group spread in small children and the elderly.

**Table 2:** Coefficient estimates from the best performing model (endemic seasonality (S2) + epidemic seasonality (S1) + trend + uncorrelated random effects)

	Estimates	CI 2.5%	CI 97.5%	Std. Error
*Epidemic Component:*				
Age [05–14]	0.041	0.024	0.072	0.012
Age [15–24]	0.064	0.040	0.101	0.015
Age [25–44]	0.044	0.024	0.080	0.014
Age [45–64]	0.114	0.063	0.206	0.034
Age [65+	1.796	1.085	2.971	0.461
Population Size	1.431	1.036	1.978	0.165
Sine (2* ***π*** *t/52)	0.929	0.871	0.992	0.032
Cosine (2* ***π*** *t/52)	0.744	0.697	0.794	0.019
Random Intercept	7.548	1.434	39.731	6.396
*Endemic Component:*				
Age [05–14]	0.180	0.092	0.350	0.061
Age [15–24]	0.115	0.058	0.227	0.040
Age [25–44]	0.136	0.071	0.263	0.046
Age [45–64]	0.154	0.080	0.297	0.052
Age [65+	0.355	0.181	0.695	0.122
Primary Schools (%)	0.869	0.657	1.149	0.142
Nurseries (%)	1.113	0.866	1.431	0.128
Hospitals (%)	0.997	0.898	1.107	0.053
Sine (2* ***π*** *t/52)	1.112	0.991	1.248	0.071
Cosine (2* ***π*** *t/52)	1.142	1.000	1.303	0.060
Sine (4* ***π*** *t/52)	0.850	0.781	0.925	0.038
Cosine (4* ***π*** *t/52)	0.830	0.771	0.894	0.019
Random Intercept	89.232	48.502	164.162	27.754
*Spatial weights (d)*	3.617	3.313	3.948	0.162
*Overdispersion*	1.337	1.305	1.370	0.012

### Norovirus reporting patterns

**Fig. 3:**
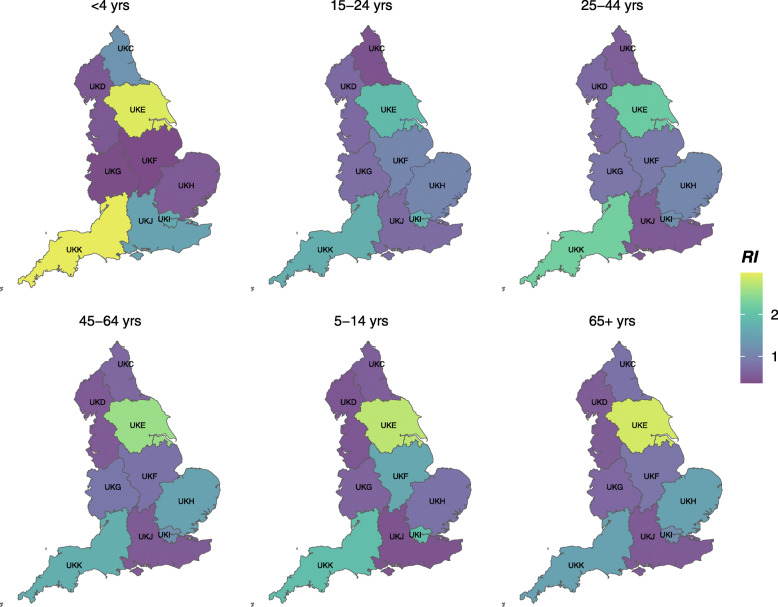
Age group- and region-specific endemic random intercepts. RI < 1 suggests lower number of norovirus reports than expected. The Nomenclature of Territorial Units for Statistics (NUTS) Level 1 codes can be mapped to regional names as follows: UKC - North East, UKD – North West, UKE – Yorkshire & Humber, UKF – East Midlands, UKG – West Midlands, UKH – East of England, UKI – London, UKJ – South East, UKK – South West

Reporting patterns were described in terms of endemic and epidemic random intercepts (RI) per age group and region (Fig. 3); values close to one and less than one imply lower number of reports than expected given the linear trend, harmonic waves, population structure, number of primary schools, nurseries and hospitals. Endemic random intercept varied from the lowest in East Midlands (UKF) in those in the under 5 year age-group (0.36, CI 0.18–0.72) to the highest in the same age group in South West (UKK)(3.00, CI 1.68–5.35) and Yorkshire & the Humber (UKE) (2.93, CI 1.74–4.94). Overall, regions displayed in purple consistently across the age groups (North West – UKD, West Midlands – UKG) are the most likely suspects for underestimation of norovirus burden (Fig. 3). In contrast, regions such as Yorkshire & the Humber and South West (UKK) are displayed in shades of green and blue.

Also, regions varied in age-related reporting patterns. For example, the lowest endemic RI for North East (UKC) was identified in those in the 15–24 age group (0.43, CI 0.18–1.04), for East Midlands (UKF) it was the young children (0.36, CI 0.18–0.72) and for South East (UKJ) it was school age children (0.44, CI 0.23–0.81). Some combinations of age groups and regions showed wide confidence interval ranges spanning from below one to over one pointing towards high levels of uncertainty, e.g. South West (UKK) in elderly (1.48, CI 0.80–2.74).

As judged by variance, the regional differences in the epidemic RI were less pronounced ($Var\left({b}_{gr}^{\left(\phi \right)}\right)=0.129$) compare to endemic RI ($Var\left({b}_{gr}^{\left(\nu \right)}\right)=0.450$). As in the endemic random intercepts, the under 5 years age group showed the highest variation across regions with the highest epidemic RI in the South West (2.49, CI 1.83–3.38; < 5 yrs) and the lowest in the West Midlands (0.44, CI 0.29–0.66; < 5 yrs). All regions were closer to the expected incidence, except South East. In the South East, across all age groups, the epidemic RI was lower than the endemic with the lowest epidemic RI in elderly (0.71, CI 0.52–0.97). North East showed a slightly different pattern in epidemic and endemic RI. The model identified an unexpectedly low number of reports in epidemic RI but only in the age-groups from 5 to 64 years. In terms of endemic RI, young children were the only group to reach a value above one. Further details are provided in the Additional files 3 and 4. The patterns of heterogeneity were stable across models in both components.

## Discussion

We aimed to describe norovirus reporting patterns in England using mixed-effect modelling. We started by describing a relationship between age-stratified weekly reports of norovirus by region and a set of predictors including seasonal waves, relevant regional covariates, spatial relationship between regions, within region norovirus activity and average contact between the age-groups. After determining the best model, we analysed endemic and epidemic random intercepts. We found that reporting practices vary greatly across regions and subpopulations, and that the seasonal changes in reporting related to differences between outbreaks and sporadic cases.

### Context

Our analysis identified geographic areas where reporting of norovirus was lower than expected, given the age structure of the population, social contacts between groups and covariates such as number of primary schools, nurseries and hospitals. As explained by Gibbons et al. [23], there are two main reasons for disease burden underestimation: 1) Under-ascertainment and 2) Under-reporting. The former occurs when community cases do not seek healthcare, and the latter when cases presenting to healthcare do not reach the surveillance system due to failure to diagnose or report a pathogen correctly. Reporting patterns described in this study capture these instances with random effects. The endemic random effects in some regions were low for all the age groups (North West and West Midlands) suggesting that under-ascertainment or under-reporting is more likely in these areas. Since norovirus is not a notifiable disease, this may lead to the perception that it is a low priority pathogen. The perception together with unavailability of a specific norovirus treatment could play a critical role when a clinician decides on whether to request a sample. For a reference laboratory reporting of an identified pathogen is mandatory through legislation [5]; therefore, here, the variability may be explained by differences in testing practices rather than reporting per se. Besides, the speed of recovery can be another factor as most of the people will recover between 12 and 72 h [3] and so they may not have contact with medical services to provide a sample during the period of illness. In contrast, for some regions, random effects were atypically high. This may in part reflect regions and laboratories that have historically functioned as sentinel surveillance centres for norovirus (for example Avon in South West as described in [24, 25]) or are very proactive in reporting (for example Yorkshire & the Humber), and to a degree may respond to particular research interests among virologists or infectious diseases specialists in the region.

Furthermore, our data suggest that the regional reporting variation was most pronounced in the sub-population of young children, who had the largest difference in reporting between the most passive and active regions in both outbreaks and sporadic cases. Thus, in some areas, norovirus in younger children could be more likely to be underestimated compared to other groups. These results agree with previous studies indicating that norovirus in children is underdiagnosed in England [26]. However, further research is needed to clarify the extent of the issue compared to other subpopulations.

The model suggests that cases from outbreaks are more likely to be reported; disproportionally higher elderly populations were shown to be associated with an increased epidemic incidence of reported norovirus infection, with a weaker association identified in the endemic sub-model. Also, the distribution of epidemic random intercepts was narrower compare to the endemic suggesting that reporting practices are relatively similar across regions and age-groups when it comes to outbreak-generated cases. The variation we see is likely related to the number of samples collected per outbreak. For example, a study investigating care homes outbreaks in North West points out that even though at least six samples are recommended, the median is only three [27]. Most of the reported cases of norovirus are epidemic in nature (86%). These findings strongly support the hypothesis that cases of norovirus from outbreaks in nursing homes and hospitals are more likely to appear in national statistics.

### What this study adds

This study adapted HHH4 analytic pipeline [11, 18, 19] to analyse norovirus reporting practices across regions and selected age groups in England. One of the benefits of this method was that it allows borrowing strength between age-groups as opposed to formulating a separate model for each group [19], some of which have low numbers of reported cases. This means that the same procedure can be followed by regional PHE units using higher spatial granularity. Additionally, we pointed out some of the biases in the otherwise stable and consistent national surveillance system, such as the tendency to see outbreak-related rather than sporadic cases in the national statistics and regional variation. Furthermore, we described the reporting patterns of norovirus, which can be of use to public health policymakers. Models facilitate focusing upon regions and subpopulations in which under-reporting may be most pronounced and have the power to highlight whether the reporting for different subpopulations within a particular region is low. Variability in reporting emphasises the importance of considering uncertainty as it has implications for decisions regarding the development of more targeted norovirus risk management (e.g. vaccine), and overall, these insights are relevant to potential norovirus forecasting efforts which are likely to follow the path of seasonal influenza, e.g. [28]. In light of this, we point out that multivariate approaches have clear benefits over separate age- or region-based models as they allow for spatial relationships. However, at the regional geographic level of granularity, the spatial effects are relatively small and so modelling every region of England on its own when age-stratification is unnecessary or unavailable could yield valid predictions as well.

### Limitations

A weakness of this analysis is that the level of geographical resolution in the data is coarse, and this limits the depth of inference. Despite the inclusion of regional factors (number of hospitals, schools and nurseries) and population structure, random effects may have captured some residual spatial variation that could potentially be explained by other factors such as genotype. However, given that routine analysis did not detect any changes or shift in the main genotypes circulating at this time [29], our analysis is unlikely to have been affected by such change. Also, our approach was not able to differentiate between under-ascertainment and under-reporting. Moreover, the POLYMOD study took place in 2005/2006, and the contact patterns in the study period could be different during the studied period. Despite these limitations, we have applied the methods to consistently collected data with the best resources available.

## Conclusion

Our findings contribute to the understanding of norovirus reporting patterns in England and provide a basis for future norovirus forecasting endeavours. There is inherent uncertainty in the routinely collected surveillance data, which needs to be recognised and methods of analysis adjusted accordingly. Regional differences were anticipated as attitudes towards the importance of norovirus surveillance as well as testing practices vary across reference laboratories and hospitals. Our analysis highlighted regions in which sporadic cases may be underestimated. Understanding the biases in surveillance data and sources of variation is crucial, especially as disease forecasting tools are increasingly developed and applied. In this context, multivariate approaches should be favoured over separate age- or region-based models. Besides forecasting, future research could enhance understanding of why under-reporting takes place, and so inform targeted norovirus risk management strategies.

## Supplementary Information

**Additional file 1.** Number of confirmed norovirus cases in England (2014/15–2018/19). Colours mark the partition of the data into training (2014 w27–2018 w26) and test periods (2018 w27–2019 w26).

**Additional file 2.** Modelling results to determine the number of seasonal waves. Comparison of fixed-effect models with one (S = 1), two (S = 2) and three (S = 3) seasonal waves in endemic component to determine the baseline model formulation.

**Additional file 3.** Epidemic and endemic random intercepts by region and age group with confidence intervals.

**Additional file 4.** Age group- and region-specific epidemic random intercepts.

## Data Availability

The dataset analysed during the current study is available from the corresponding author on reasonable request. A synthetic version of the dataset and R codes supporting the conclusions of this article are available in the GitHub repository, [10.5281/zenodo.4464124].

## Abbreviations

PHE: Public Health England
AIC: Akaike information criterion
BIC: Bayesian information criterion
SGSS: Second Generation Surveillance System
ONS: Office for National Statistics
RI: Random intercept
NUTS: Nomenclature of Territorial Units for Statistics
