# Supplementary figures and images for: Understanding norovirus reporting patterns in England: a mixed model approach
Source: BMC Public Health. 2021 Jun 28;21:1245. doi: 10.1186/s12889-021-11317-3 (PMC8240379; doi:10.1186/s12889-021-11317-3)

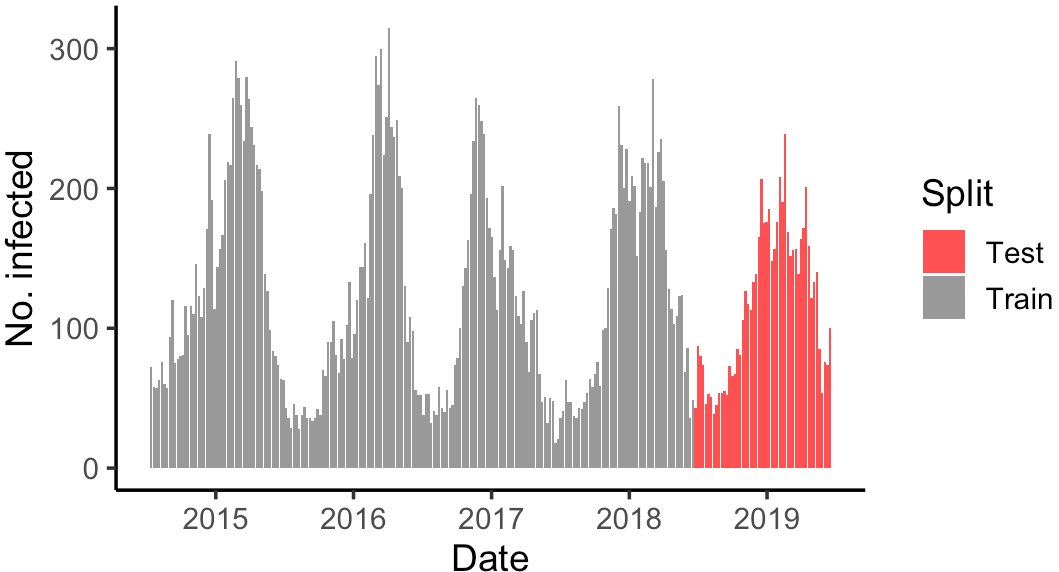

Supplement: Supplementary file 1 — Additional file 1. Number of confirmed norovirus cases in England (2014/15–2018/19). Colours mark the partition of the data into training (2014 w27–2018 w26) and test periods (2018 w27–2019 w26). [file 12889_2021_11317_MOESM1_ESM.png]

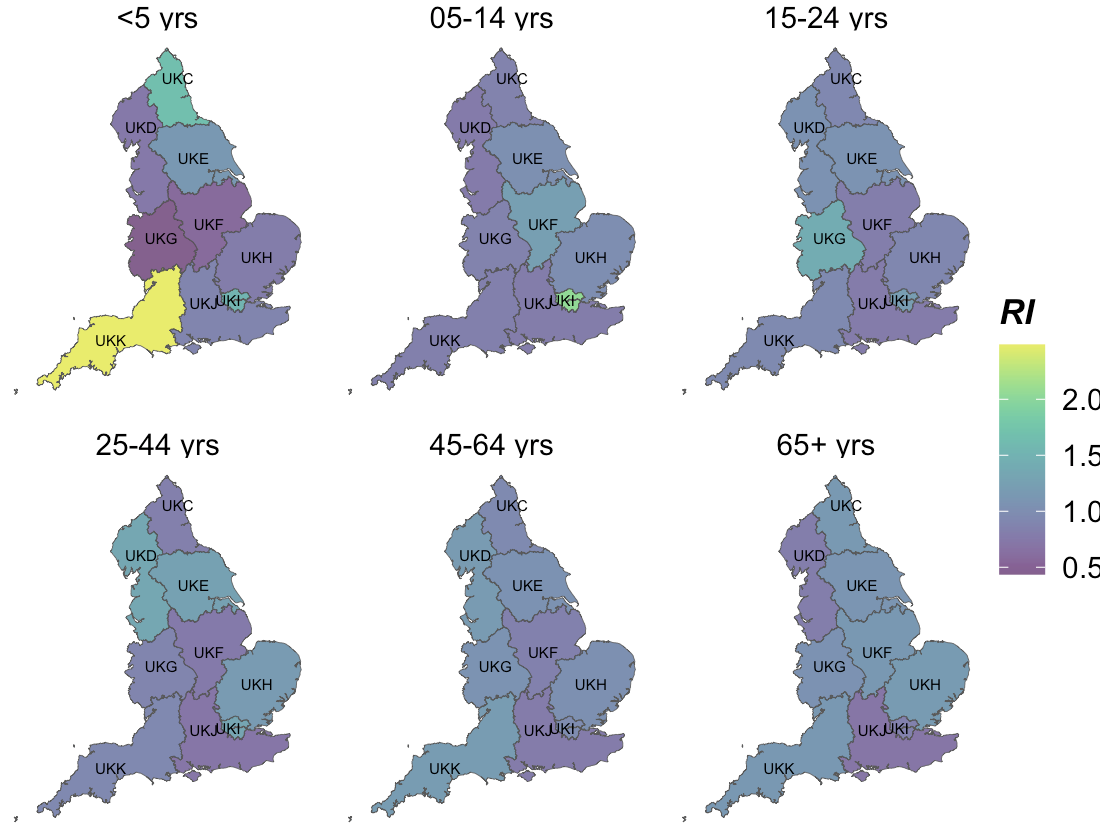

Supplement: Supplementary file 4 — Additional file 4. Age group- and region-specific epidemic random intercepts. [file 12889_2021_11317_MOESM4_ESM.png]
