# Supplementary material for: Understanding norovirus reporting patterns in England: a mixed model approach
Source: BMC Public Health. 2021 Jun 28;21:1245. doi: 10.1186/s12889-021-11317-3 (PMC8240379; doi:10.1186/s12889-021-11317-3)
Supplement: Supplementary file 2 — Additional file 2. Modelling results to determine the number of seasonal waves. Comparison of fixed-effect models with one (S = 1), two (S = 2) and three (S = 3) seasonal waves in endemic component to determine the baseline model formulation. [file 12889_2021_11317_MOESM2_ESM.docx]

[Additional file 1]

There was no difference between the fixed models with varying seasonality (S=1, S=2, S=3). There were two fixed model formulations, one without any seasonal component and one with a seasonal wave in epidemic component. The rounded scores were the same for all of the models with no seasonal component in epidemic component in terms of RPS (RPS = 0.938) and both fixed model formulations with all of the seasonal component variations achieved the same log score, logS=1.48). The RP scores and permutation tests for the fixed model with seasonal wave in epidemic component are presented in the table below.

|  | RPS | Permutation test (p-value) |
| --- | --- | --- |
| S = 1 | 0.935 | p(S = 1 vs. S = 2) = 0.389 |
| S = 2 | 0.934 | p(S = 2 vs. S = 3) = 0.443 |
| S = 3 | 0.934 |  |
